# Evaluation of the antioxidative potential of diisopropyldithiocarbamates sodium salt on diclofenac-induced toxicity in male albino rats

**DOI:** 10.1016/j.toxrep.2022.03.052

**Published:** 2022-04-09

**Authors:** Toluwani Tella, Ademuyiwa Adegbegi, Chiedu Emeninwa, Adekunle Odola, Ayansina Ayangbenro, Oluwatosin Adaramoye

**Affiliations:** a Department of Biochemistry, North-West University, Mmabatho 2735, South Africa; b Biochemistry Unit, Department of Science Laboratory Technology, Rufus Giwa Polytechnic, Owo, Ondo State, Nigeria; c Department of Biochemistry, University of Ibadan, Ibadan, Oyo State, Nigeria; d Department of Chemistry, University of Ibadan, Ibadan, Oyo State, Nigeria; e Food Security and Safety, Faculty of Natural and Agricultural Sciences, North-West University, Mmabatho 2735, South Africa

**Keywords:** CAT, Catalase, DDTC, Diethyldithiocarbamate, DIC, Diclofenac, GSH, Reduced glutathione, LPO, Lipid peroxidation, Na i-Pr_2_dtc, Diisopropyldithiocarbamate sodium salt, NC, Normal control, NSAID, Non-steroidal anti-inflammatory drug, POSS, Positive oxidative stress status, PUFA, Polyunsaturated fatty acids, ROS, Reactive oxygen species, SOD, Superoxide dismutase, VIT E, Vitamin E, Diisopropyldithiocarbamates sodium salt, Diclofenac, Antioxidant, Testes and vitamin E

## Abstract

Diclofenac (DIC) is a non-steroidal anti-inflammatory drug (NSAID) which is known to induce oxidative stress. Dithiocarbamates are compounds with proven antioxidant effect. The aim of the present study was to investigate the antioxidant capacity of diisopropyldithiocarbamates sodium salt (a synthetized compound) (Na(i-Pr_2_dtc)**)** against diclofenac-induced toxicity in the testes of male Wistar albino rats. The animals were assigned into six groups of six rats each. Group 1 (control) received corn oil, Groups 2, 3, 4, 5, 6 received DIC (100 mg/kg), DIC and (Na(i-Pr_2_dtc) (30 mg/kg), DIC and vitamin E (30 mg/kg), (Na(i-Pr_2_dtc) (30 mg/kg) and vitamin E only respectively. Our findings show that treatment with DIC significantly reduced superoxide dismutase (SOD) activity by 42% compared to normal control (NC) animals. In DIC treated group, Na(i-Pr_2_dtc) caused a 17% elevation of catalase (CAT) activity compared to DIC only group. Reduced glutathione level was significantly reduced in DIC only treated group when compared with DIC and VIT E treated group. Photomicrographs of testis from Na(i-Pr_2_dtc) treated rats showed normal seminiferous epithelium with no lesions. In conclusion, Na(i-Pr_2_dtc) has antioxidant properties.

## Highlights

• Diisopropyldithiocarbamate restored catalase and glutathione level depressed by diclofenac treatment.
• Diisopropyldithiocarbamate protected the testes in diclofenac treated rats.
• Diisopropyldithiocarbamate has antioxidant effect.

## Graphical Abstract

**Figure:**
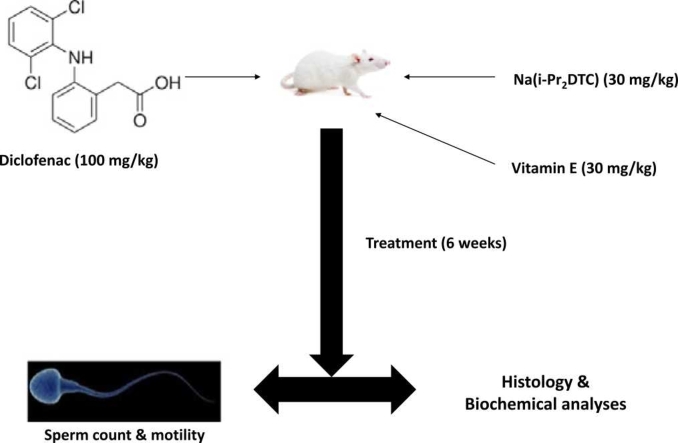

## 1 Introduction

Non-steroidal anti-inflammatory drugs (NSAIDs) are frequently used as analgesics. Diclofenac (DIC) is one of the NSAIDs with analgesic, anti-inflammatory and anti-pyretic properties [1]. Previous studies reported that DIC administration results in the production of reactive oxygen species (ROS) leading to oxidative stress [2], [3]. In addition, it has been reported that DIC is linked with hepatoxicity [4], nephrotoxicity [5], neurotoxicity [6], and reproductive toxicity [7], [45]. Due to the high level of polyunsaturated fatty acids (PUFA) of the mammalian spermatozoa, it is very susceptible to ROS attack [8], [9]. Abnormal spermatozoa have been identified as one of the etiologies for male infertility through the excessive generation of ROS. Lipid peroxidation (LPO) of sperm membrane is considered to be the key mechanism of this ROS-induced sperm damage leading to infertility [10]. Vitamin E is the most important lipid-soluble antioxidant and its main function is to protect against LPO [11].

Sodium dithiocarbamates are formed by the interaction of amines, carbon disulfide and sodium hydroxide (NaoH) [12]. The reaction of reactive nitrogen and oxygen species with dithiocarbamates generates dithiocarbamate thiyl radicals, which dimerize to form thiuram disulfides [13]. Thiuram disulfides are the oxidized form of dithiocarbamates and are responsible for the pro-oxidant effects of dithiocarbamates [14]. The antioxidant behavior of dithiocarbamates is often acknowledged with less appreciation for their pro-oxidant effects. Various studies revealed the antioxidant properties of sodium diethyldithiocarbamate (DDTC) [15], [16]. Despite the fact that several dithiocarbamate moieties have been identified and tested for various therapeutic effects, based on our findings, there is no report on the antioxidative potential of diisopropyldithiocarbamate sodium salt. Thus, diisopropyldithiocarbamate sodium salt was tested for its antioxidant properties.

The body has various mechanisms to counteract the deleterious effect of oxidative stress by endogenous and exogenous antioxidants [17]. Antioxidants can be grouped into enzymatic and non-enzymatic antioxidants and they neutralize the toxic effects produced by free radicals [18]. Biological systems have developed endogenous defense mechanisms to help protect against free radicals. Antioxidant enzymes such as catalase (CAT) and superoxide dismutase (SOD) metabolize toxic oxidative intermediates and they all require micronutrient as cofactors such as copper, zinc, selenium, manganese, iron for optimum catalytic activity [19]. In this study, antioxidant capacity of Na(i-Pr_2_dtc) is compared with a standard antioxidant (vitamin E).

## 2 Materials and methods

### 2.1 Materials

Chemicals: The chemicals used were supplied from Sigma chemical company, USA. The materials used include animals, test compound (diisopropyldithiocarbamates sodium salt**),** vitamin E, and corn oil.

### 2.2 Animals

Thirty-six (36) male Wistar albino rats (120–150 g) were procured from Covenant Farm limited, Gbolasire, Iwo Road, Ibadan, Oyo-State. The animals were put in cages and maintained under standardized environmental conditions at room temperature under controlled light cycle of 12 hr light/12 hr dark in a ventilated animal house. The animals had free access to standard rat diet (Ladokun Feeds Nig. Limited, Ibadan, Nigeria) and water ad libitum for the duration of the experiment*.* The treatment and handling of rats conform to the guidelines of Faculty of Basic Medical Sciences, University of Ibadan Animal Ethics Committee.

### 2.3 Preparation of test compound (Dithiocarbamate)

**Fig. 1:**
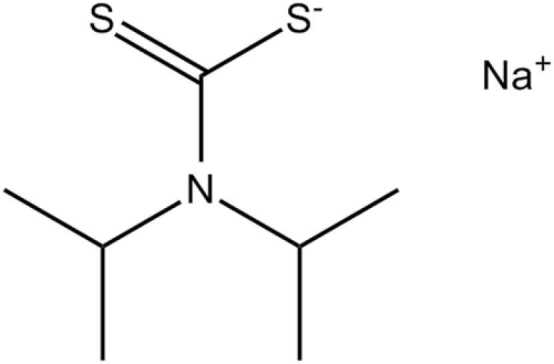
Structure of diisopropyldithiocarbamate sodium salt.

Synthesis of diisopropyldithiocarbamate sodium salt: The sodium dithiocarbamate used was produced by the interaction of carbon disulfide (CS_2_), amine and sodium hydroxide (NaoH) as described by Crovetto et al. [20] and Espigares et al. [12]. The amine HCl was dissolved in water and treated with a sufficient amount of NaOH to liberate the base and neutralize the protons generated during the production of the dithiocarbamic salt synthesis. To avoid a temperature increase, S_2_C was added in small amounts. The reaction medium was held at room temperature for 8 h while being stirred. It was then filtered and evaporated to dryness in a vacuum at a temperature of no more than 60 °C. In acetone, the solid component was dissolved, leaving a NaCl residue. To eliminate contaminants, the solution was evaporated again and treated with ethylic ether. Finally, the resulting product was recrystallized with a few drops of benzene in acetone. The structure of the test compound is presented in Fig. 1.

### 2.4 Experimental design

Thirty-six (36) rats used in this experiment were assigned into six (6) groups at the end of acclimatization period. The animals in (groups 3 and 5) and (groups 4 and 6) were pretreated with (Na(i-Pr_2_dtc) and VIT E respectively. The animals in group 1 were given corn oil, animals in groups 2, 3, 4, 5 and 6 received DIC only, DIC and (Na(i-Pr_2_dtc), DIC and VIT E, (Na(i-Pr_2_dtc) only and VIT E only respectively. Thus, 0.3 ml of dose dilution was administered to each rat via oral gavage. All the animals were euthanized after 24 h of administration of diclofenac. The epididymis and testis were immediately excised, the testes was fixed in Bouin’s solution for histopathological analysis and fresh epididymis was processed to determine the sperm counts and motility.

### 2.5 Preparation of samples for biochemical assays

The testis of each animal was excised and homogenized, and the resulting homogenate was centrifuged at 10,000 rpm, (4 °C) for 10 min to obtain the supernatant. The supernatant was collected and stored for subsequent biochemical analysis.

### 2.6 Determination of enzyme activity

#### 2.6.1 Protein determination

Protein concentration of the samples was determined by means of the Biuret method as described by Gornall et al. [21].

#### 2.6.2 Determination of catalase (CAT) activity

CAT activity was assayed by measuring the formation of chromic acetate at 570 nm as described by Sinha [22]. In a small Erlenmeyer flask, the assay mixture contained 4 ml of H_2_O_2_ solution (800 pmoles) and 5 ml of phosphate buffer (50 ml). A moderate swirling motion was used to quickly combine 1 ml of adequately diluted enzyme preparation into the reaction liquid. At room temperature, the reaction was carried out. At 60 s intervals, 1.0 ml of the reaction mixture was removed and blown into 2 ml of dichromate. The amount of hydrogen peroxide in the removed samples was measured in small test tubes (6 ml)·H_2_O_2_ was introduced together with 2 ml dichromate. When the reagent was added to H_2_O_2_, an unstable blue perchromic acid precipitate was formed almost instantly. The hue of the solution changed to a stable green after heating for 10 min in a boiling water bath due to the production of chromic acetate. The volume of the reaction mixture was produced 3 ml after cooling at room temperature, and the optical density was measured with a spectrophotometer at 570 nm.

#### 2.6.3 Determination of superoxide dismutase (SOD) activity

The SOD activity was determined by the ability of the enzyme to inhibit the autoxidation of epinephrine according to the method of Misra and Fridovich [23].

#### 2.6.4 Estimation of reduced glutathione (GSH) level

Reduced glutathione (GSH) level was estimated by measuring the rate of formation of chromophoric product resulting from the reaction of 5,5-dithiobis-2-nitrobenzoic acid and sulfhydryl compounds at 412 nm according to the method of Jollow et al. [24].

#### 2.6.5 Assessment of lipid peroxidation level

Lipid peroxidation level was determined by measuring the thiobarbituric acid reactive substances (TBARS) produced which was read at 532 nm according to the method of Rice-Evans et al. [25].

#### 2.6.6 Estimation of spermatozoa motility and count

Sperm motility was assessed according to the methods of Zemjanis [26] and Rezvanfar et al. [27]. Slicing the epididymis in 5 ml of Ham's F10 and incubation for 5 min at 37 °C in a 5% percent CO_2_ environment allowed sperm to swim out of the epididymal tubules. On a microscope slide, one drop of sperm suspension was deposited, and a cover slip was placed over the droplet. Using a phase contrast microscope, at least 10 microscopic fields were examined at 400 magnification, and the percentage of motile sperm was determined microscopically within 2–4 min of their isolation from the epididymis and expressed as a percentage of motile sperm of the total sperm counted.

The testicular spermatid counts was done by the method as described by Pant and Srivastava [28]. Briefly, the epididymis was minced in distilled water and filtered through a nylon mesh, an aliquot was placed in a haemocytometer chamber for spermatozoa head counts.

#### 2.6.7 Histology of testes

The tissues were fixed in Bouin’s solution and dehydrated in graded concentrations of ethanol. Thereafter, clearing was done in xylene and the tissue embedded in paraffin. The specimen was sectioned at 4 µm and stained with haematoxylin and eosin dye and was examined under a light microscope by a histopathologist.

#### 2.6.8 Statistical analysis

Data were analyzed using Graph Pad Prism 2007. Statistical comparison between groups was done using one way analysis of variance (ANOVA) and student t-test. Values of p < 0.05 were considered statistically significant.

## 3 Results

### 3.1 The effect of Na(i-Pr2dtc) on the body and testis weight

Table 1 shows the effect of Na(i-Pr2dtc) on the body and testis weight of the animals. There were differences in the weight between the initial and final weight of each treatment at p > 0.05.

**Table 1:** Effect of diisopropyldithiocarbamates sodium salt (Na(i-Pr_2_dtc) on the body weight and weight of testis of experimental animals.

Treatment	Initial weight (g)	Final weight (g)	Weight Difference (g)	Weight of testis (g)	Relative Weight of Testis (% body weight)
Normal control (NC)	115.0 ± 5.48	116.0 ± 4.18	1.00 ± 0.03	0.83 ± 0.13	0.71 ± 0.05
Diclofenac (DIC) only	153.0 ± 5.16	132.0 ± 14.38	-21.00 ± 0.02	1.43 ± 0.49	1.08 ± 0.04
Diclofenac and Na(i-Pr_2_dtc)	150.0 ± 0.02	102.5 ± 5.01	-47.50 ± 0.13	0.87 ± 0.11	0.85 ± 0.03
DIC and Vitamin E (VIT E)	132.0 ± 4.08	107.0 ± 5.77	-25.00 ± 0.07	0.67 ± 0.19	0.63 ± 0.05
Na(i-Pr_2_dtc) only	120.0 ± 0.01	112.5 ± 5.01	-7.50 ± 0.57	0.88 ± 0.08	0.78 ± 0.04
VIT E only	118.0 ± 11.69	116.0 ± 11.40	-2.00 ± 0.28	0.59 ± 0.17	0.51 ± 0.07

#### 3.2 Effect of Na(i-Pr_2_dtc) on SOD and CAT activities in testes

The results in Table 3 show SOD activity in the testes of male albino rats after 6 weeks of treatment. The activity of SOD was significantly (p > 0.05) raised in normal control animals when compared to DIC only treated animals. VIT E treatment returned SOD activity to near normal levels. The activity of catalase after 6 weeks of treatment is shown in Table 3. Notable was the significant increase (p > 0.05) of catalase activity in DIC and Na(i-Pr_2_dtc) treated animals compared to DIC only treated animals. The enzyme activity in DIC and Na(i-Pr_2_dtc) treated animals falling below that of DIC and VIT E treated animals. The highest amount of catalase activity was seen in DIC and VIT E treated animals.

**Table 3:** Effect of diisopropyldithiocarbamates sodium salt (Na(i-Pr_2_dtc)) on catalase (CAT) and superoxide dismutase (SOD) activity in diclofenac-treated rats.

Treatment	Catalase (CAT) (U/mg protein)	Superoxide dismutase (SOD) (U/mg protein)
Control (NC)	610.43 ± 40.53	1.47 ± 0.01
Diclofenac (DIC) only	591.91 ± 35.07	0.84 ± 0.08*
Diclofenac and Na(i-Pr_2_dtc)	690.41 ± 7.05#	0.90 ± 0.02
DIC and Vitamin E (VIT E)	710.90 ± 55.75	1.32 ± 0.03
Na(i-Pr_2_dtc) only	574.21 ± 59.09	1.14 ± 0.15
VIT E only	620.68 ± 3.89	1.41 ± 0.01

*p < 0.05 compared to NC, ^**#**^ p < 0.05 when compared to DIC.

### 3.3 Effect of Na(i-Pr_2_dtc) on GSH and lipid peroxidation levels and live/dead ratio in testes

The highest level of GSH was found in VIT E only treated group. There was increase in GSH level in DIC and VIT E treated group compared to DIC only treated group (Table 2). Na(i-Pr_2_dtc) only treated group had values similar to normal control (NC) group. The DIC only treated group had the highest amount of lipid peroxidation. There was reduction in lipid peroxidation level in DIC and Na(i-Pr_2_dtc) treated animals compared to DIC only treated animals. DIC and VIT E treated group had the lowest amount of lipid peroxidation. There is also a significant decrease (p < 0.05) in the live/dead in the DIC only treated group when compared with normal control (NC) animals (Table 2).

**Table 2:** Effects of diisopropyldithiocarbamates sodium salt (Na(i-Pr_2_dtc)) on testicular reduced glutathione (GSH), lipid peroxidation levels and live/dead ratio in diclofenac-treated rats.

Treatment	Reduced glutathione (GSH) (μg g^−1^)	Lipid peroxidation (μmol mg^–1^ protein)	Live/Dead (%)
Control (NC)	58.04 ± 0.01	7.3 ± 0.26	98 ± 0.01
Diclofenac (DIC) only	55 ± 0.01	8.0 ± 0.69	93.5 ± 5.10*
Diclofenac and Na(i-Pr_2_dtc)	56 ± 0.3	7.3 ± 0.01	95 ± 0.01
DIC and Vitamin E (VIT E)	58.25 ± 0.01	6.64 ± 0.16	95 ± 0.01
Na(i-Pr_2_dtc) only	58.25 ± 0.01	6.83 ± 0.01#	95 ± 0.01
Vitamin E (VIT E) only	60.38 ± 0.01^#^	6.82 ± 0.13	98 ± 0.01^#^

*p < 0.05 compared to NC, ^**#**^ p < 0.05 when compared to DIC.

### 3.4 Effect of Na(i-Pr_2_dtc) on sperm count and motility

**Fig. 2:**
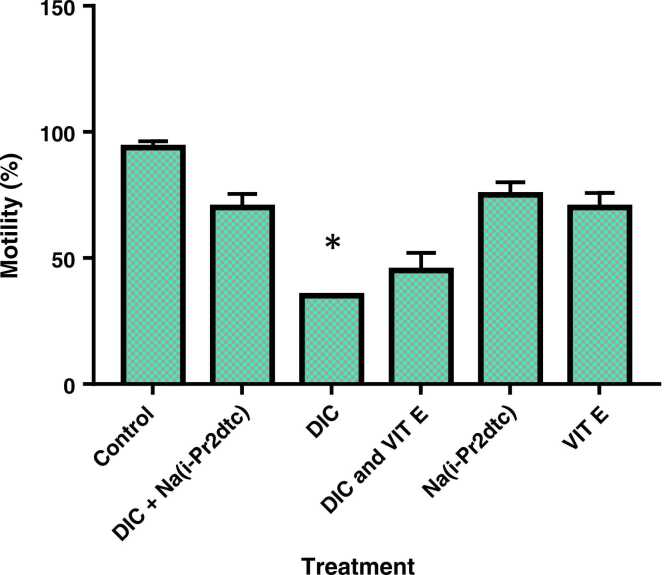
Effect of diisopropyldithiocarbamates sodium salt (Na(i-Pr_**2**_dtc)) on sperm motility. * p < 0.05 compared to normal control (NC).

**Fig. 3:**
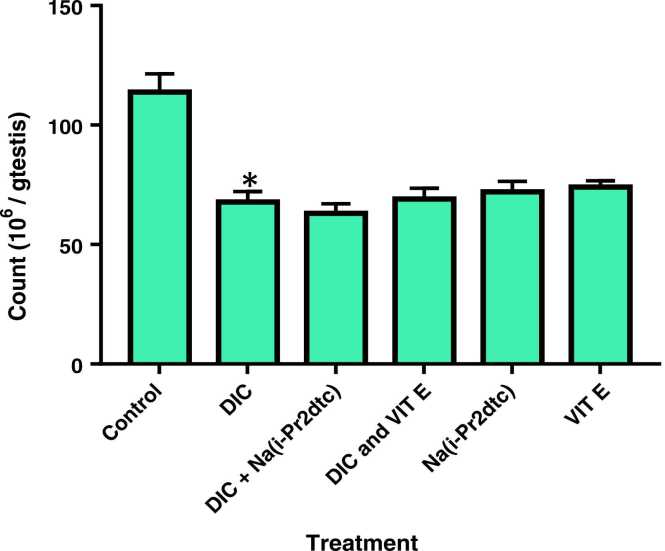
Sperm count in the testes of rats after 6 weeks of treatment. Data is represented as mean ± SD. * p < 0.05 compared to normal control (NC).

The results in Fig. 2, Fig. 3 show that there was a significant (p < 0.05) decrease in sperm count and motility in the DIC with Na(i-Pr_2_dtc) treated group when compared with DIC-only treated group.

### 3.5 Effect of Na(i-Pr_2_dtc) on the histological structure of the testes

**Fig. 4:**
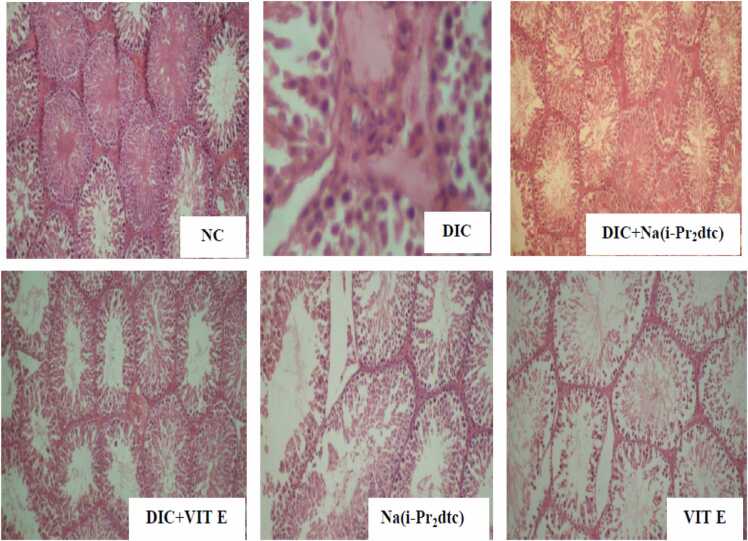
Photomicrographs of sections of the testes from normal (NC), diclofenac (DIC), vitamin E (VIT E) and diisopropyldithiocarbamate sodium salt (Na(i-Pr2dtc) treated rats.

In the normal control animals, the tissue showed normal histological structure with no visible lesions, while DIC treatment caused the formation of interstitial oedema ( Fig. 4). In the DIC and Na(i-Pr_2_dtc) treated animals, there was mild congestion of seminiferous epithelium. The seminiferous tubules in the Na(i-Pr_2_dtc) only treated animals appear normal when compared with DIC only treated group (Fig. 4).

## 4 Discussion

The purpose of this study is to evaluate the toxicity of DIC and the antioxidative potential of Na(i-Pr_2_dtc). Our findings revealed that Na(i-Pr_2_dtc) ameliorated the toxic effects diclofenac caused in rats. Comparative analysis was done using a standard antioxidant (vitamin E). Enzymes such as CAT, SOD and non-enzymatic parameter like GSH [29], [30], [31], which act as ROS scavengers are contained in large amount in spermatozoa and seminal plasma as well as a variety of substances with SOD or CAT like activities [32]. The marked decrease in CAT and GSH concentration in the testes after DIC administration was previously reported [33], this is considered to be due to diclofenac-induced toxicity in the testes. It has been proved from the results of this study that while diclofenac causes toxicity in the reproductive system studied in this experiment, treatment with Na(i-Pr_2_dtc) restored enzymatic activities and this could be attributed to the antioxidant properties of Na(i-Pr_2_dtc). In line with our findings, Vyas et al. [7] reported that diclofenac treatment adversely affected the normal histological structure of the testes, causing degenerations in germ cells, interstitial cells and seminiferous tubules.

In human reproduction, when ROS production exceeds critical levels, this can overwhelm antioxidant defense strategies of spermatozoa causing oxidative stress [34]. ROS attacks DNA, leading to strand breaks and oxidative base damage in human spermatozoa [35]. Due to the high concentration of polyunsaturated fatty acids (PUFA) of the mammalian spermatozoa, it is very susceptible to ROS attack which results in a decrease in sperm viability and motility and increased morphological defects [36]. Sun et al. [37] observed negative correlation between DNA fragmentation and semen quality as reflected by sperm count, motility and morphology. Malondialdehyde, one of the byproducts of lipid peroxidation has been used in biochemical assays to evaluate the degree of peroxidative damage sustained by spermatozoa [38], the results of such an assay exhibit an excellent correlation with the degree to which sperm function is impaired in terms of motility [39]. Evidence suggests that dithiocarbamates significantly prevented increase in lipid peroxidation concentration in the testes [40]. Our results show that Na(i-Pr_2_dtc) significantly reduced lipid peroxidation level in Na(i-Pr_2_dtc) treated only animals compared to DIC only treated animals. Vyas and colleagues [7] reported that sperm count and motility were reduced in a dose-dependent manner after DIC treatment. Adeyemi et al. [33] reported that chronic administration of DIC triggers oxidative stress. Positive oxidative stress status (POSS) occurs when there is a shift towards pro-oxidants, because of either reduced level of antioxidants or excess ROS. In DIC and Na(i-Pr_2_dtc) treated animals, POSS might account for the observed decline in sperm count and motility, increased dosage and duration of Na(i-Pr_2_dtc) might improve sperm count and motility. There is evidence that taking antioxidant supplements such as GSH, vitamin E and vitamin C improve semen quality [41], [42], [43]. A study in Indian industrial workers suggested that taking 1000 mg of vitamin C fives times a week for 3 months significantly improved sperm count and motility [44]. Our findings show that Na(i-Pr_2_dtc) has antioxidant effect and that the effect might be related to dose and duration of administration.

In conclusion, our findings confirmed the antioxidant role of Na(i-Pr_2_dtc) in abating diclofenac-induced oxidative damage of testes in rats and to the best of our knowledge this is the first report on the antioxidant properties of the diisopropyldithiocarbamate.

## Authors statement

We declare that this manuscript is original and has not been published before. We confirm that the manuscript has been read and approved by all named authors, and the order of authors listed in the manuscript has been approved by all of us. We understand that the Corresponding Author is responsible for communicating with the other authors about progress and submissions of revisions.

## Declaration of Competing Interest

The authors declare that they have no known competing financial interests or personal relationships that could have appeared to influence the work reported in this paper.
